# Epigenetic roles of KDM3B and KDM3C in tumorigenesis and their therapeutic implications

**DOI:** 10.1038/s41419-024-06850-z

**Published:** 2024-06-26

**Authors:** Jung Yoo, Go Woon Kim, Yu Hyun Jeon, Sang Wu Lee, So Hee Kwon

**Affiliations:** College of Pharmacy, Yonsei Institute of Pharmaceutical Sciences, Yonsei University, Incheon, 21983 Republic of Korea

**Keywords:** Cancer epigenetics, Diagnostic markers, DNA

## Abstract

Advances in functional studies on epigenetic regulators have disclosed the vital roles played by diverse histone lysine demethylases (KDMs), ranging from normal development to tumorigenesis. Most of the KDMs are Jumonji C domain-containing (JMJD) proteins. Many of these KDMs remove methyl groups from histone tails to regulate gene transcription. There are more than 30 known KDM proteins, which fall into different subfamilies. Of the many KDM subfamilies, KDM3 (JMJD1) proteins specifically remove dimethyl and monomethyl marks from lysine 9 on histone H3 and other non-histone proteins. Dysregulation of KDM3 proteins leads to infertility, obesity, metabolic syndromes, heart diseases, and cancers. Among the KDM3 proteins, KDM3A has been largely studied in cancers. However, despite a number of studies pointing out their importance in tumorigenesis, KDM3B and KDM3C are relatively overlooked. KDM3B and KDM3C show context-dependent functions, showing pro- or anti-tumorigenic abilities in different cancers. Thus, this review provides a thorough understanding of the involvement of KDM3B and KDMC in oncology that should be helpful in determining the role of KDM3 proteins in preclinical studies for development of novel pharmacological methods to overcome cancer.

## Facts

- KDM3 proteins regulate a vast number of genes crucial for proper development and growth, and their malfunction leads to various physiological diseases.
- Although KDM3B and KDM3C fall under the same protein family, KDM3B and KDM3C are evolutionarily quite distant from KDM3A and each other.
- KDM3B and KDM3C are aberrantly expressed in different cancers, implying their potential as therapeutic targets to overcome cancer.

## Open Questions

- What are the ways to develop specific inhibitors of individual KDM3 proteins that do not target the conserved sequences or domains of KDM3 proteins?
- What are the molecular mechanisms of oncogenic signaling pathways that KDM3 proteins are involved in, such as the Hippo pathway, Wnt pathway, and Notch pathway?

## Introduction

**Fig. 1:**
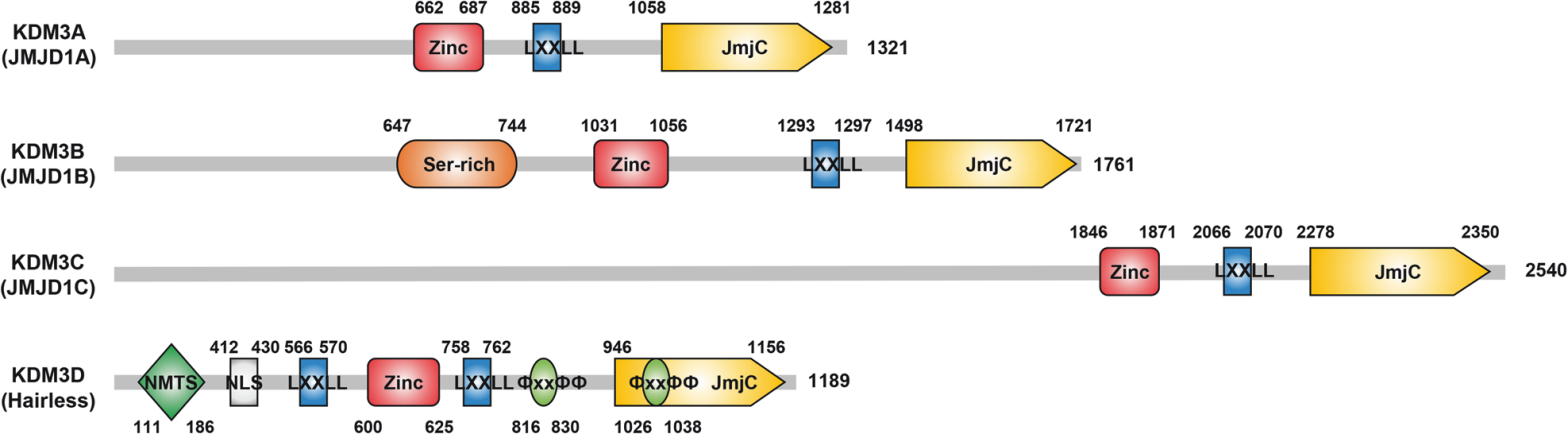
Schematic representation of domains of individual lysine demethylase 3 (KDM3) proteins. KDM3A (NP_001140160.1) consists of 1321 amino acids, KDM3B (NP_057688.3) contains 1761 amino acids, KDM3C (NP_116165.1) has 2540 amino acids, and KDM3D (NP_005135.2) has 1189 amino acids. Red domains represent zinc-finger-like domains, blue domains represent leucin-rich domains (LXXLL), and yellow domains represent jumonji-C (JmjC) domains. The other domains include serine-rich domain represented with orange (Ser-rich), nuclear matrix targeting signal (NMTS) represented by green, nuclear localization signal (NLS) represented by gray, and thyroid hormone receptor interaction domains (ϕXXϕϕ) represented by light green.

Ever since the discovery of lysine demethylases (KDMs), extensive studies have demonstrated that their deregulation is frequently observed in various cancers. KDMs have been identified as tumor suppressors or oncogenes in numerous types of malignant cancers. Of the many KDM subfamilies, KDM3 family proteins encompass four members: KDM3A, KDM3B, KDM3C, and KDM3D. Generally, only KDM3A, KDM3B, and KDM3C are regarded as members of the KDM3 subfamily because KDM3D is evolutionarily quite distant from the other three proteins [1]. Domains of KDM3A-D are displayed in Fig. 1, detailed descriptions are mentioned elsewhere [2]. KDM3 proteins regulate gene expression largely by removing mono- or di-methyl groups on histone H3 lysine 9 (H3K9me1/me2).

KDM3 family proteins show pleiotropic biological functions. Knockout of *Kdm3a* increases H3K9me1/me2. *Kdm3a* complete knockout mice are viable but exhibit reversed male-to-female sex phenotype due to transcriptional defects of the sex-determining gene *Sry* of the Y chromosome, which is responsible for the differentiation of the bipotential gonads into testes [3]. Also, both *Kdm3a*-mutant and *Kdm3a*-depleted mice display male infertility and impaired spermatogenesis with smaller testis sizes and decreased sperm count, indicating that KDM3A plays a key role in adult testes [4, 5]. In addition to its reproductive role, Kdm3a is required for homeostasis, thermogenesis, and energy metabolism. For instance, *Kdm3a* knockout mice cause adult obesity phenotype, but no other additional phenotypes [5–7]. Similar to *Kdm3a*, *Kdm3b-*knockout male mice are viable but subfertile due to defects in spermatogenesis and male sexual behaviors, such as mounts, intromissions, and ejaculation, without alteration in the global levels of H3K9 methylation in the testis [8]. However, *Kdm3b* female knockout mice exhibit irregular estrous cycles with decreased ovulation, fertilization, and uterine decidual response, and are therefore infertile [9]. In addition*, Kdm3b* knockout mice have decreased levels of insulin growth factor binding protein-3 expression in the kidney and in the blood, resulting in postnatal somatic growth retardation. Knockout of *Kdm3b* hinders H3K9me2 and/or H4R3me2 demethylation at distinct gene clusters and impairs activation of genes essential for hematopoietic stem and progenitor cells (HSPC) survival, differentiation, and development [10]. As a result, *Kdm3b* knockout mice show defects in hematopoiesis. In the same sense, *Kdm3*c constitutive knockout mice show defects in male gametogenesis, preweaning lethality [11], mydriasis, and homeotic transformation of the vertebrae [12]. *Kdm3d* (also known as *hairless*) knockout mice exhibit congenital hair loss and defects in epidermis proliferation and differentiation, resulting in severe wrinkling of the skin [13, 14]. Thus, KDM3D controls epithelial cell differentiation period in both the epidermis and hair follicles.

**Fig. 2:**
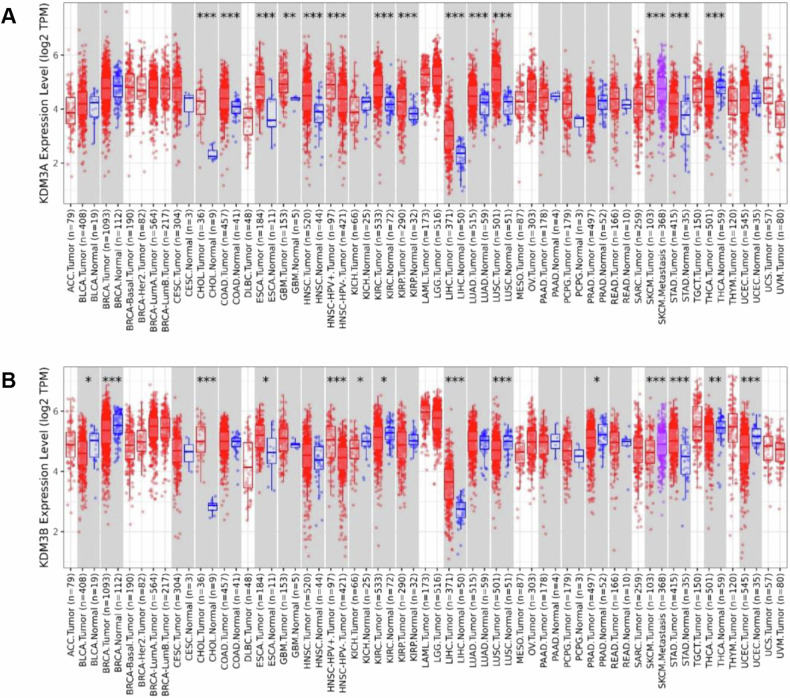
Expression levels of KDM3 in different cancers. **A** Different expression patterns of KDM3A in various cancers. **B** Different expression patterns of KDM3B in various cancers. Distributions of gene expression levels are displayed using box plots. Data were analyzed with the Wilcoxon test and its significance is annotated by the number of stars. *p < 0.05, **p < 0.01, ***p < 0.001.

KDM3 proteins exert prominent roles in normal development, and their malfunction may lead to heart diseases, intellectual disabilities, and obesity [1]. Mounting studies have also identified that dysregulation of KDM3 is responsible for tumorigenesis in breast cancer, colon cancer, lung cancer, ovarian cancer, and prostate cancer [2]. An online database analysis (TIMER 2.0) shows the differential expression patterns of KDM3A and KDM3B (KDM3C not available) in various cancers and adjacent normal tissues across all TCGA tumors, highlighting their importance in tumorigenesis (Fig. 2). While a predominant number of research reports on the significant role of KDM3A in various cancers, an increasing number of studies imply aberrant activities of KDM3B and KDM3C and their deregulation in a variety of cancers. Hence this review specifically addresses the roles of KDM3B and KDM3C in cancers.

## The role of KDM3B in tumorigenesis

KDM3B (JMJD1B; JHDM2B; 5qNCA) demethylates both H3K9me1/me2 and H4R3me2s (symmetric dimethylarginine) substrates with similar efficiency in vitro to activate gene expression [15]. However, KDM3B distinctly regulates H3K9me2 and H4R3me2s demethylation at different loci in vivo. Interestingly, KDM3B has been observed to simultaneously demethylate H3K9me2 and H3R3me2s in certain groups of genes, while it may demethylate only H3K9me2 or only H3R3me2 in other groups of genes. More studies are required to define the biochemical properties behind such differences.

Unlike KDM3A, which is mostly regarded as an oncogene [2], KDM3B is the first KDM3 to be proposed as a tumor suppressor gene in acute myeloid leukemia (AML), colorectal cancer (CRC), and breast cancer (BCa) (16-19)] (Table 1). Although these findings support KDM3B as a tumor suppressor, it also shows oncogenic functions as it is overexpressed in prostate cancer (PCa) [16], non-small cell lung carcinoma (NSCLC) [17], hepatocellular carcinoma (HCC) [18], renal cell carcinoma [19], and acute lymphoblastic leukemia (ALL) [20], promoting cancer cell proliferation and survival. Therefore, these findings suggest the tumor-suppressing or oncogenic activities of KDM3B may rely on its specific interacting protein partners and target genes in a different cellular context (Table 2).

**Table 1:** Different levels and possible functions of KDM3 in tumorigenesis.

Cancer	Protein levels and tumorigenic functions of:		
	KDM3A	KDM3B	KDM3C
Breast cancer	Overexpressed; oncogene [68]	Downregulated; tumor suppressor [27] Overexpressed; oncogene [28]	Tumor suppressor [50]
Bladder cancer	Overexpressed; oncogene [69]	N/A	N/A
Colorectal cancer	Overexpressed; oncogene [70]	Downregulated; tumor suppressor [29] Overexpressed; oncogene [32]	Overexpressed; oncogene [54]
Esophageal cancer	N/A	N/A	Overexpressed; oncogene [44, 55]
Leukemia	N/A	Downregulated; tumor suppressor [21, 22, 71] Overexpressed; oncogene [20]	Oncogene [41, 47, 48, 51–53, 58, 60]
Lung cancer	Overexpressed; oncogene [72]	Overexpressed; oncogene [17]	N/A
Liver cancer	Overexpressed; oncogene [73]	Overexpressed; oncogene [18, 33]	N/A
Kidney cancer	N/A	De novo mutation [19]	N/A
Ovarian cancer	Overexpressed; oncogene [74]	N/A	N/A
Pancreatic cancer	Overexpressed; oncogene [75]	N/A	N/A
Prostate cancer	Overexpressed; oncogene [76]	Overexpressed; oncogene [16]	Reduced [56] Oncogene [57]

*N/A* not available.

**Table 2:** Interacting partners and functions of KDM3 proteins.

Protein	Interacting partner	Function	References
KDM3A	Androgen receptor (AR)	Coactivator of AR	[76, 77]
	Hypoxia-inducible factor 1α (HIF-1α)	Coactivator of HIF-1α	[78, 79]
	Lysine demethylase 4B	Cooperates to regulate estrogen receptor (ER) target genes	[80]
	Non-receptor tyrosine kinase (ACK1 or TNK2)	Phosphorylation of KDM3A stimulates ER-regulated *HOXA1* transcription	[64]
	Brahma related gene 1 (BRG1)	Promotes BRG1 activity on MUC1 promoter	[72, 81]
	p53	Removes K372me on p53 to inhibit p53 transcriptional activity	[82]
	β-catenin	Enhances transcriptional activity of β-catenin	[70]
	p300	Required for p300 recruitment to target genes	[83]
KDM3B	β-catenin	Enhances transcriptional activity of β-catenin	[70]
	CREB-binding protein (CBP)	Recruited to *LIM Domain Only 2* (*LMO2*) promoter	[20]
	YTHDC1	removing H3K9me2 on the promoter of *FAM111A*	[34, 35]
KDM3C	RUNX1-RUNX1T1 (AML1-ETO)	Coactivator of RUNX1-RUNX1T1	[41]
	Homeobox A9 (HOXA9)	Unclear	[48]
	Ring Finger Protein 8 (RNF 8)	Stabilized by RNF8 after DNA damage	[50]
	AR	Coactivator of AR	[61]

*YTHDC1* YTH N^6^-methyladenosine RNA binding protein.

### Acute lymphoblastic leukemia (ALL) and acute myeloid leukemia (AML)

**Fig. 3:**
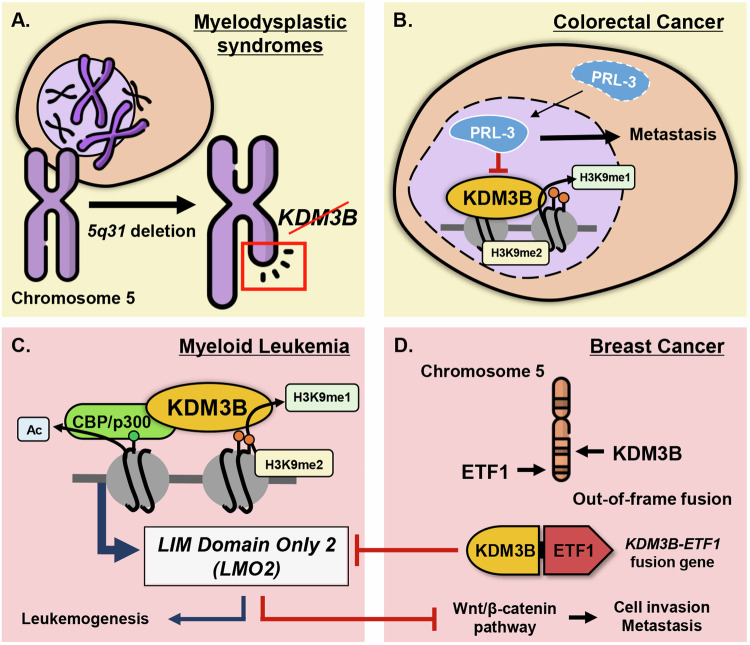
Oncogenic and tumor suppressive functions of KDM3B in tumorigenesis. **A** KDM3B is in the *5q31* chromosome region, which is frequently deleted in myelodysplastic syndromes. **B** Nuclear phosphatase of regenerating liver 3 (PRL-3) is negatively correlated with KDM3B in colorectal cancer. PRL-3 localizes to the nucleus and inhibits KDM3B activity, leading to metastasis of colorectal cancer cells. **C** KDM3B interacts with histone acetyltransferase CBP/p300 to induce expression of leukemic oncogene, LIM Domain Only (LMO2). **D** KDM3B-Eukaryotic translation termination factor (ETF1) fusion gene in breast cancer inhibits the expression of LMO2. Inhibition of LMO2 leads to increased Wnt/β-catenin pathway, leading to breast cancer cell invasion and metastasis.

KDM3B was first reported as a tumor-suppressor gene against hematopoietic malignancies [21]. In myelodysplastic syndromes (MDS) and AML, KDM3B is located at the frequently deleted *5q31* chromosome region (Fig. 3A). Additionally, KDM3B knockout mice show abnormal phenotypes in the hematopoietic system like in MDS patients, including granulocytosis, leukocytosis, and mild anemia [15]. Indeed, KDM3B is downregulated in AML patients and KDM3B overexpression inhibits colony formation of *5q31* deleted AML cells [22]. Furthermore, *Homeobox A1 (HOXA1)* was identified as a target gene for KDM3B in AML. *HOXA1* is generally known as an oncogene in many solid tumors, but Xu and colleagues state that it may have a tumor-suppressive role in AML. KDM3B binds to the retinoic acid response element (RARE) of *HOXA1* and induces its expression by removing H3K9me2 on its RARE. Indeed, KDM3B-depletion reduced *HOXA1* levels in various AML cells. In line with this, KDM3B overexpression repressed colony formation of AML cells, implicating the tumor-suppressive role of KDM3B in AML.

Unlike its level in AML, KDM3B was found to be overexpressed in ALL-type leukemia patients [20]. During leukemic transformation, KDM3B functions as a transcriptional coactivator and interacts with histone acetyltransferase CBP, which acetylates H3/H4. Simultaneous demethylation of H3K9 and acetylation on H3/H4 by KDM3B and CBP, respectively, stimulate target gene expression, such as a leukemic oncogene *LIM Domain Only 2* (*LMO2*), during leukemogenesis (Fig. 3C). Furthermore, analysis of leukemia patient tissues showed that KDM3B levels were increased in ALL-type leukemia patients than in AML-type leukemia patients. Consistently, *LMO2* expression level was higher in ALL-type patient samples than that of AML-type patients and normal samples. In all, KDM3B is reported to carry tumor-suppressive in AML, yet oncogenic properties in ALL. Such seemingly ambivalent roles of KDM3B may be context-dependent for leukemia, and more studies are required to verify its role in leukemic tumorigenesis.

### Prostate cancer (PCa)

Proper functioning of KDM3B is required for normal spermatogenesis in vivo [8]. As a result, abnormal alterations to KDM3B expression may point to PCa development. Indeed, an extensive analysis of the mRNA expression level of all 32 histone demethylases in PCa confirmed that KDM3B is significantly increased in PCa samples compared to those of the normal prostate [16]. Another study also showed that KDM3B is crucial for castration-resistant prostate cancer (CRPC) proliferation and survival [23]. As androgen receptor (AR) signaling is crucial in all stages of PCa progression, as PCa patients are usually treated with androgen deprivation therapy [24]. However, almost all the PCa patients develop a more aggressive type of PCa, known as CRPC [25]. Of the various KDMs, KDM3A interacts with AR and regulates AR-target gene expression [26]. Unlike KDM3A, KDM3B does not affect CRPC growth by changing the canonical AR pathway or cell cycle pathways [23]. KDM3B knockdown in LNCaP-abl cells, a model of androgen-independent CRPC, did not show significant alterations in the levels of AR-regulated genes. Instead, a reduction in the proliferation of CRPC cells by KDM3B was associated with metabolic changes. *Arginase 2* and *retinol dehydrogenase 11*, which are both highly expressed metabolic proteins in healthy prostate tissues, were downregulated upon KDM3B knockdown. Also, KDM3B knockout in LNCaP-abl cells showed decreased levels of several amino acids, such as histidine and tyrosine, that are known to be important in PCa growth. A metabolomics analysis comparing blood samples obtained from patients with PCa and benign prostatic hyperplasia showed that PCa patients had elevated levels of several metabolites, including histidine and tyrosine. Although these studies demonstrate the role of KDM3B in PCa development, further research is necessary in order to see it as a novel pharmacological target.

### Breast cancer (BCa)

Contradicting studies demonstrate the different roles of KDM3B in BCa progression. One study highlights that high expression of KDM3B suppresses BCa tumorigenesis [27]. Oncomine database shows that KDM3B is upregulated in normal breast tissues compared to BCa tissues, suggesting its role as a tumor suppressor. Gene expression based data obtained from GOBO online database confirms that low expression of KDM3B in BCa patients correlates with shorter relapse-free survival in the lymph node-negative tumors. Contrary to the implicated tumor-suppressive role of KDM3B in BCa, another study has identified a possible oncogenic role of KDM3B in BCa progression [28]. Hu and colleagues have found that a novel KDM3B-Eukaryotic translation termination factor 1 (ETF1) fusion gene is highly expressed in BCa cells, playing a major role in BCa cell invasion and metastasis in vitro and in vivo. KDM3B-ETF1 inhibits the expression of *LMO2*, activating the Wnt/β-catenin signaling pathway in BCa (Fig. 3D). The contradiction between the two findings could be attributed to the change of function in the fusion gene when KDM3B combines with ETF1. Nonetheless, more research needs to be conducted to understand the exact function of KDM3B in BCa.

### Colorectal cancer (CRC)

KDM3B plays vital tumor-suppressive roles in CRC. In CRC, KDM3B is downregulated by a key metastasis gene, *phosphatase of regenerating liver 3* (*PRL-3)* [29] (Fig. 3B). PRL-3 is a phosphatase involved in the progression and metastasis of several types of cancers, including CRC [30, 31]. In advanced-stage CRC patients, nuclear PRL-3 is increased while cytoplasmic PRL-3 is decreased. Such nuclear translocation of PRL-3 promotes CRC cell invasion and metastasis [29]. Nuclear PRL-3 reduces the demethylase activity of KDM3B, disturbing the H3K9 methylation state in CRC. In clinical samples, KDM3B expression level is lower in most of the primary CRC tissue samples compared to their normal counterparts. Also, expression of KDM3B is inversely correlated with lymph node status, Dukes’ classification, and tumor staging. However, further studies are inevitable to solidify the exact mechanism nuclear PRL-3 regulates the activity of KDM3B. Despite the tumor suppressive role associated with PRL-3, KDM3B has also been reported to contribute to CRC recurrence and chemoresistance via activating the Wnt signaling pathway [32]. Simultaneous knockdown of KDM3A and KDM3B reduced the tumorigenic potential of cancer stem-like cells in CRC cells through regulation of the Wnt/β-catenin pathway. Both genes are recruited to Wnt target gene promoters to activate Wnt target gene transcription by removing H3K9me2. All these results highlight the need to verify the functions of KDM3B as a tumor suppressor or an oncogene in CRCs, as it may be a potential therapeutic target for CRC treatment.

### Lung cancer

KDM3B is significantly upregulated and is a noteworthy contributor to poor recurrence-free survival in NSCLC [17]. Poor recurrence-free survival in NSCLC is mostly attributed to development of resistance to chemotherapeutic drugs and strategies, such as the standard taxane-platin combination therapy. Evaluation of preclinical resistance samples showed that KDM3B levels are higher in taxane-platin-chemoresistant NSCLC cells compared to corresponding parental cells. Indeed, KDM3B is progressively upregulated throughout the resistant series and shows increased expression in the resistant model in vivo. Moreover, taxane-platin-resistant cells and tumors develop hypersensitivity to pan-KDM inhibitor, JIB-04, and KDM5/KDM6 inhibitor, GSK-J4, in vitro and in vivo. Furthermore, these KDM inhibitors partially reverse deregulated transcriptional programs in taxane-platin-resistant cells and synergize with standard chemotherapy in vitro and in vivo. Although the above KDM inhibitors are not KDM3B-specific, these results provide insight into the possibility that pharmacological targeting of KDM3B in chemoresistant NSCLC cells could be a novel therapeutic strategy for lung cancer therapy and chemoresistant tumors.

### Liver cancer

Analysis of publicly available RNA-sequencing and clinical tissue sample data shows that KDM3B is overexpressed in liver tissues of HCC patients compared to the matched normal tissues [18, 33]. Knockout of KDM3B hampers cell cycle progression and proliferation of HCC cells [18]. About 30% of the KDM3B knockout cells show chromosome instability, such as mitotic spindle multipolarity, and cell death via cell cycle delay in polynucleated cells. Also, KDM3B depletion significantly downregulates the expression of cell cycle-related genes, like *cyclin D1*, and cell proliferation factors, like *CDC123*. KDM3B is recruited by *N*^6^-methyladenosine (m^6^A) reader, YTHDC1, to m^6^A-associated chromatin regions. Recruited KDM3B contributes to HCC progression by removing H3K9me2 on the promoter of *FAM111A* [34, 35]. FAM111A, a single-stranded DNA-binding serine protease that promotes DNA synthesis, is reported to be increased in HCC and correlates with poor survival of HCC patients. Contrary to the proposed oncogenic role of KDM3B in hepatocarcinogenesis, another study suggests otherwise. Inhibition of KDM3B expression in HCC by miR-1307 promotes hepatocarcinogenesis by enhancing the expression of endoplasmic-reticulum-related gene, *CALR*, disrupting the normal functioning of endoplasmic-reticulum [36]. While other studies highlight the role of KDM3B in HCC, one study undermines the role of KDM3B in HCC tumorigenesis, as there seems to be no significant correlation between KDM3B expression levels and HCC patient survival rates [33]. These results indicate that further studies need to be conducted to solidify the function of KDM3B in HCC.

### Kidney cancer

*KDM3B* is one of the genes contributing to a childhood kidney cancer called Wilms tumor. Analyzing exome sequencing of lymphocyte DNA from Wilms tumor involving 890 individuals has shown that *TRIM28, FBXW7, KDM3B*, and *NYNRIN* are new predisposition genes of Wilms tumor [19]. Based on the exome data of possible childhood predisposition genes, two de novo mutations (Asn1141Ser, 916_917delAG) of *KDM3B* have been found in a child with Wilms tumor. Another study identified other de novo *KDM3B* mutations in AML and Hodgkin lymphoma patients [37]. This large exome sequencing study suggests that *KDM3B* variants may be important features of Wilms tumor.

## The role of KDM3C in tumorigenesis

KDM3C (JMJD1C; JHDM2C; TRIP8) was first identified in a yeast two-hybrid assay as thyroid receptor-interacting protein 8 (TRIP8) [38, 39]. Human *KDM3C* mRNA is expressed in diffuse-type gastric cancer, insulinoma pancreatic islet, and undifferentiated embryonic stem cells [40]. Although KDM3C has originally been reported as an H3K9me2/me1 demethylase-inducing transcriptional activation, subsequent independent studies show conflicting conclusions that either succeeded [41–46] or failed [11, 47–50] to substantiate the existence of demethylase activity in KDM3C. Most, if not all, studies failed to prove the ability of KDM3C to remove H3K9 in vitro. In KDM3A, amino acid threonine 667 at the zinc-finger domain contributes to the H3K9me1/2 substrate specificity [49]. However, this residue is not conserved at the corresponding position in KDM3C, which could be one of the reasons for the uncertainty in the demethylase activity of KDM3C. Despite the controversies regarding the presence of demethylase activities of KDM3C, dysregulation of KDM3C is still crucial to tumorigenesis of many cancers. KDM3C is mainly reported to be required for the survival of AML [41, 47, 48, 51–53], and it is also known to function as an oncogene in CRC [54] and esophageal cancer (EC) [44, 55]. However, a few studies also report on the role of KDM3C as a tumor suppressor in PCa [56, 57] and BCa [50]. Although the tumorigenic function of KDM3C is relatively less studied compared to other KDM3 proteins, we collectively discuss the recent understandings of KDM3C in different cancers.

### Acute myeloid leukemia (AML)

**Fig. 4:**
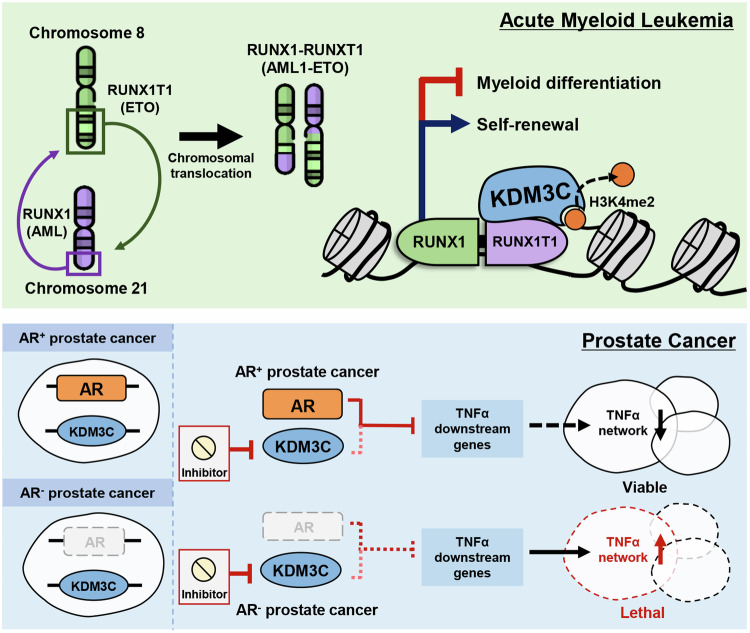
Oncogenic and tumor suppressive functions of KDM3C in tumorigenesis. Upper: In acute myeloid leukemia (AML), chromosomal translocation occurs between chromosome 8 and chromosome 21, producing a RUNX1-RUNX1T1 transcription factor. RUNX1-RUNX1T1 interacts with KDM3C and maintains low H3K9me2 levels, inducing transcription of self-renewal genes while inhibiting that of myeloid differentiation genes. Lower: Androgen receptor (AR) and KDM3C display a synthetic lethal relationship. AR and KDM3C inhibit expression of tumor necrosis factor-α (TNF-α) target genes, allowing proliferation of prostate cancer cells. KDM3C depletion in AR-positive cells does not affect cancer cell viability, but depletion of KDM3C in AR-negative prostate cancer cells is lethal.

Like KDM3B, KDM3C has been reported to be essential for carcinogenesis of AML [41, 52, 53, 58]. Through shRNA screening in AML1-ETO, MLL-AF9-, and HOXA9-driven AML cells, KDM3C was identified as a potential oncogene [47, 48]. KDM3C is required for the survival of multiple AML cell lines [41]. For example, *Mixed Lineage Leukemia* rearranged (*MLL*r) AML cell lines are sensitive to depletion of KDM3C [51]. Inhibition of KDM3C using a small molecular inhibitor, JDI-10, represses lipid synthesis genes including *FADS2*, *SCD, HMGCS1*, and *SQLE* in *MLL*r AML [58]. Suppression of such lipid synthesis genes could be the molecular mechanism behind increased apoptosis and repressed cell proliferation upon JDI-10 treatment in *MLLr* AML cells. Also, KDM3C functions as a coactivator for RUNX1-RUNX1T1 (formerly AML1-ETO) transcription factor, a product of the chromosomal translocation t(8;21) in AML [41] (Fig. 4). RUNX1-RUNX1T1 enhances self-renewal and inhibits myeloid differentiation in leukemia stem cells. It recruits KDM3C to its target genes to maintain low H3K9me2 levels, regulating target gene expression. Additionally, gene ontology analysis indicates that KDM3C-regulated genes in Kasumi-1 AML cells are enriched in genes encoding proteins that facilitate the transduction of the tyrosine kinase signaling pathway, cell proliferation, and cell differentiation. The knockdown of KDM3C decreases the number of leukemia-initiating cells by promoting differentiation and impairing cell growth, thereby accounting for the failure of leukemia establishment in serial transplantation experiments. Moreover, KDM3C is required for self-renewal in leukemia, as it functions with aberrantly expressed self-renewal-associated genes, such as *homeobox A9* (*HOXA9*). KDM3C is an essential partner of HOXA9 and therefore affects HOXA9-driven gene expression (e.g., *ALDH1L2*, *NDRG1*, and *WDFY1*) in leukemogenesis [48]. Furthermore, leukemia co-expressing KDM3C and HOXA9 contributes to tumor aggressiveness by demethylase-independent upregulation of glycolytic and oxidative metabolism [53]. A recent study strengthens the credibility of KDM3C as a potential oncogene in myeloid leukemia, as KDM3C knockdown enhances the chemotherapy sensitivity of myeloid cells [59]. Altogether, although KDM3C is vital for the maintenance of AML, it is nonessential for leukemia initiation, most likely due to the absence of demethylase activity. Because depletion of KDM3C causes minor defects in normal hematopoiesis [48], disrupting its activity with small-molecule inhibitors may be advantageous in AML-type patients [58, 60], outweighing the minimal adverse effects caused upon inhibition.

### Prostate cancer (PCa)

Unlike the other overexpressed KDM3 family members, KDM3C is found to be abnormally reduced in PCa [56]. It is well-known that a splice variant of KDM3C interacts with AR in PCa [56, 61]. A recent study by Yoshihama and colleagues demonstrates a synthetic lethal relationship between AR and KDM3C [57] (Fig. 4). KDM3C depletion suppresses the growth of AR-negative PCa cells, in vitro and in vivo. Depletion of AR and KDM3C upregulates the tumor necrosis factor-α downstream signaling pathway, both independently and in combination. KDM3C functions as one of the DNA repair factors in PCa, regulating the balance between the homologous recombination and nonhomologous end-joining repair pathways [56]. Although not as crucial as the loss of other DNA damage checkpoint proteins such as 53BP1 and Rev7, reduction of KDM3C disrupts the balance between the two DNA repair pathways, breaching the anti-cancer barrier of prostate cells. Furthermore, analysis of PCa patient tissue samples showed that KDM3C level decreased as the disease progressed. Kurfurstova and colleagues found that decreased KDM3C level is relevant to resistance to Poly (ADP-Ribose) Polymerase-1 inhibitors (PARPi) that target DNA repair pathway in hormone-receptor defective tumors, which are otherwise responsive to PARPi. In support of this finding, KDM3C has also been found to play important roles in DNA repair pathway in other cancers, like BCa [50]. KDM3C regulates the ring finger protein 8 (RNF8)- and breast cancer type 1-mediated chromatin response to DNA double-strand breaks (DSB). KDM3C erases methylation on lysine 45 of the mediator of DNA damage checkpoint 1 (MDC1), thereby promoting MDC1-RNF8 interaction and RNF8-dependent MDC1 ubiquitination for DSB repair. Without KDM3C, RNF8-mediated polyubiquitination of MDC1 is blocked, so chromatin response to DSB is impaired and hence overall maintenance of genome integrity is disturbed. As a result, KDM3C-deficient human cancer cells show enhanced resistance to irradiation and PARPi. These results substantiate the idea that monitoring KDM3C status could be a predictive biomarker in oncology and also a potential target to overcome PARPi resistance in PCa and other cancers.

### Colorectal cancer (CRC)

Contrary to its reduced level in PCa, KDM3C is relatively overexpressed in colon cancer tissues compared to the normal colon tissues, promoting tumor migration and invasion both in vitro and in vivo [54]. GEO database shows that increased KDM3C positively correlates with lymph node metastasis and poorer overall survival. Moreover, knockdown of KDM3C attenuates migration and invasion ability. Additionally, in KDM3C knockdown cells, mRNA level of *ATF2*, a transcriptional factor that binds to cAMP-responsive element and interacts with components of the Wnt pathway, is significantly suppressed compared to that of metastatic cells. Upon KDM3C depletion, global H3K9me2 level increased while H3K9me1 and H3K9me3 levels did not change, indicating that KDM3C transcriptionally regulates ATF2 via its demethylase activity. Consistently, xenograft models injected with KDM3C-depleted SW48 cells have lower lung weights as a result of lower metastasis incidences. The above results exhibit that KDM3C stands as a promising therapeutic target to overcome CRCs, but further validation is required to fully understand its oncogenic role in CRC tumorigenesis.

### Esophageal cancer (EC)

EC is one of the most lethal cancers known. KDM3C is overexpressed in EC patient tissues and EC cell lines where it positively correlates with yes-associated protein 1 (YAP1) [44]. The depletion of KDM3C robustly inhibits EC cell growth and proliferation and downregulates the YAP1 expression in both mRNA and protein levels via modulating H3K9me2 levels. Qu et al. also emphasized the important role of KDM3C in EC. They found that KDM3C is upregulated by circular RNA, circ_0006168. Knockdown of circ_0006168 inhibits tumor growth both in vitro and in vivo even in taxol-resistant EC cells [55]. However, clearly there is a need for further research to understand how KDM3C functions as an oncogene in EC.

## Discussion

The significance of lysine demethylases in tumorigenesis has been clearly recognized. Of the different KDMs, recent progresses focusing on the aberrant functional roles of KDM3 proteins in various cancers highlight their potential as biomarkers and as novel targets in treating cancers. Although much is studied about the roles of KDM3A in cancers compared to those of KDM3B and KDM3C, the latter two lysine demethylases are not to be overlooked. However, more studies are required to fill in the missing gaps in the biochemical mechanisms and roles of KDM3B and KDM3C in tumorigenesis. First, despite being a demethylase, it is uncertain whether KDM3C removes methyl groups from histone proteins as the other KDM3 proteins do [49]. To figure out whether KDM3C is capable of removing histone methylation marks, more studies need to confirm whether the demethylase activity of KDM3 proteins on H3K9me1/2 is completely dependent on the conserved amino acid found in KDM3A and KDM3B. Regardless of the presence of H3K9me1/2 demethylase ability, KDM3C still functions as an important protein by demethylating and interacting with other non-histone proteins, such as MDC1 and RNF8, respectively [50]. It has also been found that demethylase activity of KDM3C is regulated posttranslationally via phosphorylation [62], suggesting the possible reason behind the context-dependent demethylase activity of KDM3C. Upstream regulation of KDM3C by other proteins is not an odd phenomenon, as KDM3A has also been found to be regulated by phosphorylation [7, 63, 64]. Regulating the kinases or phosphatases that function on KDM3 proteins may be another option for overcoming cancer with dysregulated KDM3.

Second, more studies need to verify whether the sequence motif differences between KDM3B and KDM3C may account for their differential roles in tumorigenesis. KDM3B and KDM3C fall under the same protein family, yet KDM3B and KDM3C are evolutionarily quite distant from KDM3A and each other. The identity level at the amino acid sequence level for KDM3A and KDM3B is 59.64%, while for KDM3A and KDM3C the level decreases to 45.37%. Moreover, the identity level drops to less than half, 47.88%, when comparing KDM3B and KDM3C [1]. Interestingly, KDM3C is the most distant from the other two KDM3 proteins, which may be another reason for the unsettled discussion of whether KDM3C actually possesses a demethylase activity.

**Fig. 5:**
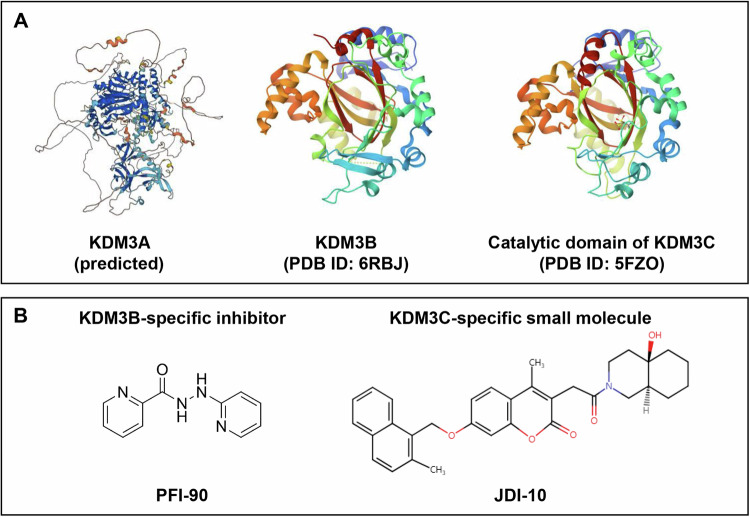
Molecular structure of KDM3 proteins and chemical structures of KDM3B and KDM3C inhibitors. **A** Experimentally determined protein structures of KDM3A, KDM3B, and KDM3C are shown. Unlike KDM3B and KDM3C had experimentally determined protein structures obtained from PDB, KDM3A had only a prediction of its protein structure obtained from Alphafold. **B** Chemical structures of KDM3B- and KDM3C-specific inhibitors, PFI-90 and JDI-10, are shown, respectively.

Third, lack of KDM3B-specific and KDM3C-specific inhibitors makes it difficult to confirm their exact biochemical roles in oncology. 5-carboxy-8-hydroxyquinoline (IOX1) is a well-known broad-spectrum inhibitor of 2-oxoglutarate-dependent-oxygenases, including other KDMs, showing the strongest inhibitory effect against KDM3A [65]. PFI-90 has been developed as a selective inhibitor of KDM3B (patent no. WO2021101929), but it still needs to be further validated whether its inhibitory activity is specific for KDM3B and be tested for its anti-tumor activity. Newly identified small molecular inhibitors of KDM3C, JDI-10 and JDM-7 repress leukemia progression in vitro [58, 60]. Along with these KDM1C inhibitors, JDI-4, JDI-12, and JDI-16 are small molecular modulators discovered by the same group that bind and inhibit both KDM3B and KDM3C [52]. However, further confirmation in vitro and in vivo is essential to validate the specific inhibitory activities of the inhibitors listed above. Lack of KDM3A/B/C-specific inhibitor is attributable to the insufficient understanding of individual KDM3 protein structures. Figure 5 shows the currently discovered experimental structures of KDM3B (PDB ID: 4C8D) and catalytic domain of KDM3C (PDB ID: 5FZO), obtained from protein data bank (PDB) [66]. Despite the number of functional studies on KDM3A in tumorigenesis, protein structure of KDM3A is yet to be determined; currently only predicted structure of KDM3A is available (Alphafold). Discovering crystal structures of KDM3 proteins would allow for a better understanding of the basis of their substrate specificity and accelerate the development of KDM3-specific inhibitors.

Lastly, deeper understanding of the cellular context-dependent roles of KDM3B and KDM3C in various cancers may provide novel insights on targeting KDM3B and KDM3C in cancer therapy. As discussed previously, KDM3B and KDM3C can function as oncogenes or tumor suppressors depending on different cancers types or other cellular conditions. Such functional variances and context-dependencies could be appertained to a few reasons. Firstly, KDM3B and KDM3C proteins may regulate genes that are tissue-specifically expressed such as AR, whose expression is largely confined to the smooth muscle tissues of the male reproductive organs [67]. Secondly, KDM3B and KDM3C have been found to function with fusion oncogenes that occur only in certain cancers. For example, KDM3B-ETF1 fusion oncogene is frequently observed in BCa, while RUNX1-RUNX1T1 fusion oncogenic transcription factor is expressed in AML. Distinctive functions KDM3B and KDM3C play in tumorigenesis could be explained by cancer-specific expression of their epigenetic target genes. Lastly, functional outcomes of KDM3B and KDM3C are also affected by the different interacting partners in the process of tumorigenesis. Various protein-protein interactions and cross-talks between different pathways decide the fate of KDM3B and KDM3C proteins to function as oncogenes or tumor suppressors. Many unresolved questions surrounding the mechanisms of KDM3B and KDM3C in tumorigenesis remain to be disclosed, leaving hope for further pharmacological opportunities to fight cancer using epigenetic therapy.
